# Evaluation of setting kinetics, mechanical strength, ion release, and cytotoxicity of high-strength glass ionomer cement contained elastomeric micelles

**DOI:** 10.1186/s12903-024-04468-3

**Published:** 2024-06-20

**Authors:** Nitchakarn Leenutaphong, Prathip Phantumvanit, Anne M. Young, Piyaphong Panpisut

**Affiliations:** 1 Faculty of Dentistry, Thammasat University, Pathum Thani, 12120 Thailand; 2 Division of Biomaterials and Tissue Engineering, UCL Eastman Dental Institute, Royal Free Hospital, Rowland Hill Street, London, NW3 2PF UK; 3 Thammasat University Research Unit in Dental and Bone Substitute Biomaterials, Thammasat University, Pathum Thani, 12120 Thailand

**Keywords:** Glass Ionomer cements, Elastomeric micelles, Mechanical properties, Ion release, Setting kinetic

## Abstract

**Background:**

Low mechanical properties are the main limitation of glass ionomer cements (GICs). The incorporation of elastomeric micelles is expected to enhance the strength of GICs without detrimentally affecting their physical properties and biocompatibility. This study compared the chemical and mechanical properties, as well as the cytotoxicity, of elastomeric micelles-containing glass ionomer cement (DeltaFil, DT) with commonly used materials, including EQUIA Forte Fil (EF), Fuji IX GP Extra (F9), and Ketac Molar (KT).

**Method:**

Powder particles of GICs were examined with SEM-EDX. Setting kinetics were assessed using ATR-FTIR. Biaxial flexural strength/modulus and Vickers surface microhardness were measured after immersion in water for 24 h and 4 weeks. The release of F, Al, Sr, and P in water over 8 weeks was analyzed using a fluoride-specific electrode and ICP-OES. The toxicity of the material extract on mouse fibroblasts was also evaluated.

**Results:**

High fluoride levels in the powder were detected with EF and F9. DT demonstrated an initial delay followed by a faster acid reaction compared to other cements, suggesting an improved snap set. DT also exhibited superior flexural strength than other materials at both 24 h and 4 weeks but lower surface microhardness (*p* < 0.05). EF and F9 showed higher release of F, Al, and P than DT and KT. There was no statistically significant difference in fibroblast viability among the tested materials (*p* > 0.05).

**Conclusions:**

Elastomeric micelles-containing glass ionomer cement (DT) exhibited satisfactory mechanical properties and cytocompatibility compared with other materials. DT could, therefore, potentially be considered an alternative high-strength GIC for load-bearing restorations.

## Background

The phasing down of dental amalgam, as part of the Minamata Convention, led to the need for alternative direct restorative materials [1]. Resin composites and glass ionomer cements (GICs) were suggested as good candidates [2]. Several studies reported satisfactory success with high-viscosity GICs for single or two-surface posterior restorations [3–5]. GICs were also reported to be a cost-effective restorative material [6–9]. The attractive properties of GICs include fluoride release, self-adhesion, and preventive effects on secondary caries by releasing multiple ions such as fluoride, strontium, and phosphate [10].

The setting mechanism of GICs is an acid-base reaction [11]. This reaction involves the neutralizing polyacrylic acids in the liquid phase by ions released from fluoroaluminosilicate glass in the powder phase, forming a network of polyacrylate salts [12]. The low flexural strength is a significant limitation of the GICs, as material fracture is one of the primary causes of failure in GIC restorations [13]. The reported flexural strength of conventional GICs at 24 h post-setting was approximately 18–34 MPa [14]. These values are substantially lower than those of resin composite, which generally exhibit flexural strength higher than 100 MPa [15, 16]. Another major concern is the low wear resistance of GICs [17]. A clinical study indicated that the clinical performance of GICs substantially decreased after 6 years due to deterioration of occlusal contour and wear [13]. A negative correlation between wear and surface microhardness of GICs has also been reported [18].

Various strategies have been proposed to enhance the mechanical strength and abrasive resistance of glass ionomer cements. One approach was metal reinforcement by incorporating metal power or silver-tin alloys into the material [19]. However, the metal-reinforced GICs failed to demonstrate an enhancement in material strength and exhibited no clinical benefits, mainly due to their poor esthetics [19, 20]. Another strategy involved incorporating reactive glass fibers. It was observed that adding these fibers (600 μm in length) at a concentration of 20 vol% approximately doubled the strength of a GIC [19]. Despite promising results, such methods have not been widely adopted in commercial materials. A recent alternative method for enhancing the mechanical strength involves incorporating polyethylene glycol (PEG) and polyurethane (PU) nanoparticles into GIC (Deltafil, DMG, Hamburg, Germany) [21]. These particles will form PEG-PU micelles with elastic structures. The ductile particles in brittle GICs were expected to help disperse energy at crack tips and form bridging zones to delay crack propagation [22].

Previous studies reported that Deltafil exhibited higher fracture toughness than other conventional GICs [21, 23]. The material was also found to demonstrate superior longevity compared to Ketac Universal and Fuji IX GP Extra upon the chewing simulation of occlusal restoration (Class I cavity) [21]. Deltafil exhibited lower abrasion loss or wear than other GICs [21], suggesting its potential suitability for load-bearing restorations. Despite these beneficial effects, the reports on the chemical, mechanical, and cytotoxic properties of GICs containing PEG-PU micelles are limited.

This study, therefore, aims to compare the setting reaction, biaxial flexural strength, surface microhardness, ion release, and cytotoxicity of GIC-containing elastomeric micelles (Deltafil) with other commonly used high-viscosity conventional GICs. The null hypothesis was that Deltafil would not exhibit significant differences in these properties when compared to other commercial GICs.

## Methods

### Characterization of GICs

Four commercial high-viscosity glass ionomer cements were used in the current study (Table 1). The characteristics of glass fillers and elemental analysis were performed using a scanning electron microscope (SEM, JSM 7800 F, JEOL Ltd., Tokyo, Japan) equipped with an energy-dispersive X-ray spectrometer (EDX, X-Max 20, Oxford Instruments, Abingdon, UK). The powder of GICs was coated with Au (Q150R ES, Quorum Technologies, East Sussex, UK) using a current of 23 mA for 45 s. The test used a beam voltage of 10 kV and the working distance set at 10 mm. The single-point EDX analysis was performed in 3 areas of the filler particle.

**Table 1:** Composition of conventional glass ionomer cements used in the current study

Materials	Composition	Powder-to-liquid ratio	Lot number	Suppliers
Deltafil (DT)	Powder: fluoroaluminosilicate glass, polyacrylic acid Liquid: polyacrylic acid, tartaric acid, PEG-PU micelles, water	4.9:1	242330	DMG, Hamburg, Germany
EQUIA Forte HT Fill (EF)	Powder: 92–97% fluoroalumino-silicate glass, 3–8% polyacrylic acid, pigment trace Liquid: 34–45% polyacrylic acid, 5–10% polybasic carboxylic acid, 45–55% water	3.0:1	22087171	GC, Tokyo, Japan
Fuji IX GP Extra (F9)	Powder: 95% fluoroalumino-silicate glass, 5% polyacrylic acid Liquid: 40% polyacrylic acid, 5–10% polybasic carboxylic acid, 50% water	3.6:1	2104131	GC, Tokyo, Japan
Ketac molar (KT)	Powder: 85–95% oxide glass, 1-6% copolymer of acrylic acid-maleic acid Liquid: 35–55% copolymer of acrylic acid-maleic acid, 40–55% water, 5–10% tartaric acid	3.4:1	8325000	3 M ESPE, St. Paul, MN, USA

### Setting kinetics

The setting kinetics of GICs were determined using attenuated total reflectance-Fourier transform infrared spectroscopy (ATR-FTIR, Nicolet iS5, Thermo Fisher Scientific, Waltham, MA, USA) (*n* = 3). Immediately after 10 s of mixing using an amalgamator (CapMix, 3 M ESPE, St. Paul, MN, USA), the materials were injected into the diamond of the ATR. Then, the FTIR spectra (400–4000 cm^–1^) were recorded from the bottom surface every 30 s for 10 min using a resolution of 4 cm^–1^. The test was performed at 25 °C. Key peak assignments for each chemical group are provided in Table 2. Peak loss and gain during the acid/base reaction were obtained by subtracting the initial spectra from those at later times to provide different spectra.

**Table 2:** Peak assignments for reactive acidic and product salt groups in GICs [24, 25]

Wave number (cm^–1^)	Assignment
1255	Acid C-O stretch
1410	Salt sym. COO^–^
1467	Salt sym. COO^–^
1554	Salt asym. COO^–^
1588	Salt asym. COO^–^
1705	Acid C-O stretch

The reaction extent (RE) of acid neutralization reactions (degree of acid-base neutralization) was determined based on the reduction of the peak representing the acid group according to the method used in a previous study [25].

1 $$RE=100* \frac{{A}_{t}-{A}_{0}}{{A}_{f}-{A}_{0}}$$

where A_0_ and A_t_ were the absorbance at 1714 cm^–1^ initially and at time, t, respectively. The final absorbance (A_f_) at infinite time (i.e., when 1/t = 0) was determined from the intercept on the y-axis of plots of late-time absorbance versus 1/t.

### Biaxial flexural strength (BFS) and modulus (BFM)

For biaxial flexural strength and modulus determination (*n* = 10), the GICs were mixed and loaded into a metal circlip (10 mm internal diameter and 1 mm in thickness). The discs were covered with an acetate sheet and glass slide. They were left at room temperature for 1 h and subsequently removed from the circlip and placed in 5 mL of deionized water. The specimens were kept in an incubator at 37ºC for 24 h or 4 weeks, then removed and placed in a ball-on-ring testing jig. They were loaded using a 500 N load cell and a crosshead speed of 1 mm/min. The failure load was recorded, and then biaxial flexural strength (BFS) and modulus (BFM) were calculated using the following equations [26].

2 $$\text{B}\text{F}\text{S} = \frac{\text{F}}{{\text{d}}^{2}}\left\{\left(1 + \text{v}\right)\left[0.485\text{l}\text{n}\left(\frac{\text{r}}{\text{d}}\right) + 0.52\right] + 0.48\right\}$$

3 $$\text{B}\text{F}\text{M} = \left(\frac{{\Delta }\text{H}}{{\Delta }{\text{W}}_{\text{c}}}\right)\times \left(\frac{{{\beta }}_{\text{c}}{d}^{2}}{{\text{q}}^{3}}\right)$$

where F is the failure load (N), d is the thickness of the disc specimens (m), r is the radius of the circular support of the ball-on-ring testing jig (m), and v is Poisson’s ratio (0.3) [26]. Additionally, $\frac{{\Delta }\text{H}}{{\Delta }{\text{W}}_{\text{c}}}$ represents the rate of change of the load about the central deflection or gradient of force versus the displacement curve (N/m) [27]. ${{\upbeta }}_{\text{c}}$ and q are the center deflection function (0.5024) and the ratio of the support radius to the specimen radius, respectively. The fracture surface of the representative tested specimen from each material was determined using SEM-EDX using a similar protocol to the first section. The EDX single-point analysis was performed from 3 areas on the fracture surface.

### Surface microhardness

For surface microhardness determination, disc specimens (10 mm in diameter and 1 mm in thickness) were prepared similarly to the previous section (*n* = 5). The specimens were immersed in 5 mL of deionized water. The Vickers surface microhardness of the specimens after immersion for 24 h was then recorded using a Vickers microhardness tester (FM-800, Future-Tech, Kanagawa, Japan). The measurement was performed using a load of 300 g with an indentation time of 10 s [28]. The result was averaged from 4 areas and expressed as Vickers hardness number (VHN). The specimens were then placed in the same solution, and the Vickers surface microhardness of the same specimens was then repeated at 4 weeks.

### Ion release

Fluoride release in deionized water from disc specimens (*n* = 5) was assessed using a fluoride-specific electrode (Orion Versastar Pro, Thermo Fisher Scientific, Waltham, MA, USA). Fluoride concentrations of 1, 10, 100, and 1000 ppm were used for calibration. Disc specimens (10 mm in diameter and 1 mm in thickness) were prepared similarly to the previous section. They were immersed in a tube containing 5 mL of deionized water. The specimens were kept in an incubator with a controlled temperature of 37ºC for up to 8 weeks. At various time points (1, 2, 3, 4, 5 days and 1, 2, 3, 4, 5, 6, 7, and 8 weeks), the specimens were removed and placed in a fresh solution. The storage solution from each time point was mixed with TISABIII using a 1:10 volume ratio. The concentration of fluoride was then measured.

For Al, P, and Sr release, another set of disc specimens (*n* = 5) was prepared and immersed in tubes containing 5 mL of deionized water. The tubes were kept at 37ºC for 8 weeks; then, the specimens were removed, and the storage solution was collected. The calibration standard was performed using an Environmental Standard (CPAchem, Bogomilovo, Bulgaria). The concentration of each element was determined using inductively coupled plasma optical emission spectrometry (ICP-OES, Optima 8300, PerkinElmer, Waltham, MA, USA). The detection range of Al, P, and Sr were 0.1–50 mg/L, 0.5–50 mg/L, and 0.1–20 mg/L, respectively.

### Cytotoxicity

Cytotoxicity testing of the extracts from disc specimens was conducted following a protocol reported in previous studies [29, 30]. Disc specimens (10 mm in diameter and 1 mm in thickness) were prepared and sterilized by 30-min UV irradiation on each of their top and bottom surfaces (*n* = 5). The specimens were then immersed in 200 µL of Dulbecco’s modified Eagle medium (DMEM, Gibco, Thermo Fisher Scientific, Grand Island, NY, USA) with 10% FBS (Gibco), 1% penicillin/streptomycin (Gibco) and 1% L-glutamine (Gibco) added. The specimens were left for 5 h at room temperature. Then, 50 µL of the medium was pipette mixed with an equal volume of fresh medium for a two-fold dilution and subsequently transferred to 96-well plates. These plates were seeded with mouse fibroblast L292 cells at a density of 8 × 10^3^ cells/well, with plain culture medium serving as the blank control.

The cells were cultured at 37 °C in a humidified atmosphere containing 5% CO_2_ for 72 h. Then, the MTT solution (0.5 mg/mL) (Invitrogen, Thermo Fisher Scientific, Grand Island, NY, USA) was added to each well for 30 min. The reaction was terminated by 100 µL of dimethylsulfoxide (Sigma‒Aldrich, St. Louis, MO, USA). The final product’s color was quantified by measuring the absorbance at 570 and 650 nm (OD, optical density) using a microplate reader spectrophotometer (Varioskan LUX Multimode, Thermo Fisher Scientific, Grand Island, NY, USA). The relative cell viability (%) was calculated according to the following Eqs. [31, 32]. The assay was conducted in triplicate.

4 $$Relative\,cell\,viability= \frac{OD\,of\,the\,test\,group}{OD\,of\,the\,control}\times 100$$

### Statistical analysis

Results for quantitative analysis, including absorbance change, BFS/BFM, surface microhardness, ion release, and cytotoxicity. These data were presented as mean and 95%CI. Data were analyzed using Prism for macOS version 10.1.1 (GraphPad Software, San Diego, CA, USA). The normality of data was checked using the Shapiro-Wilk test. Then, the results between groups were compared using one-way ANOVA followed by Tukey post-hoc multiple comparisons. Power analysis was performed using G*power version 3.1.9.6 (Heinrich Heine University Düsseldorf, Düsseldorf, Germany) [33]. The effect size was calculated using the results from the previous studies [34, 35]. These estimations suggested that the sample size used in each test would provide power greater than 0.95 at an alpha level of 0.05 for one-way ANOVA.

## Results

### Characterization of GICs

**Fig. 1:**
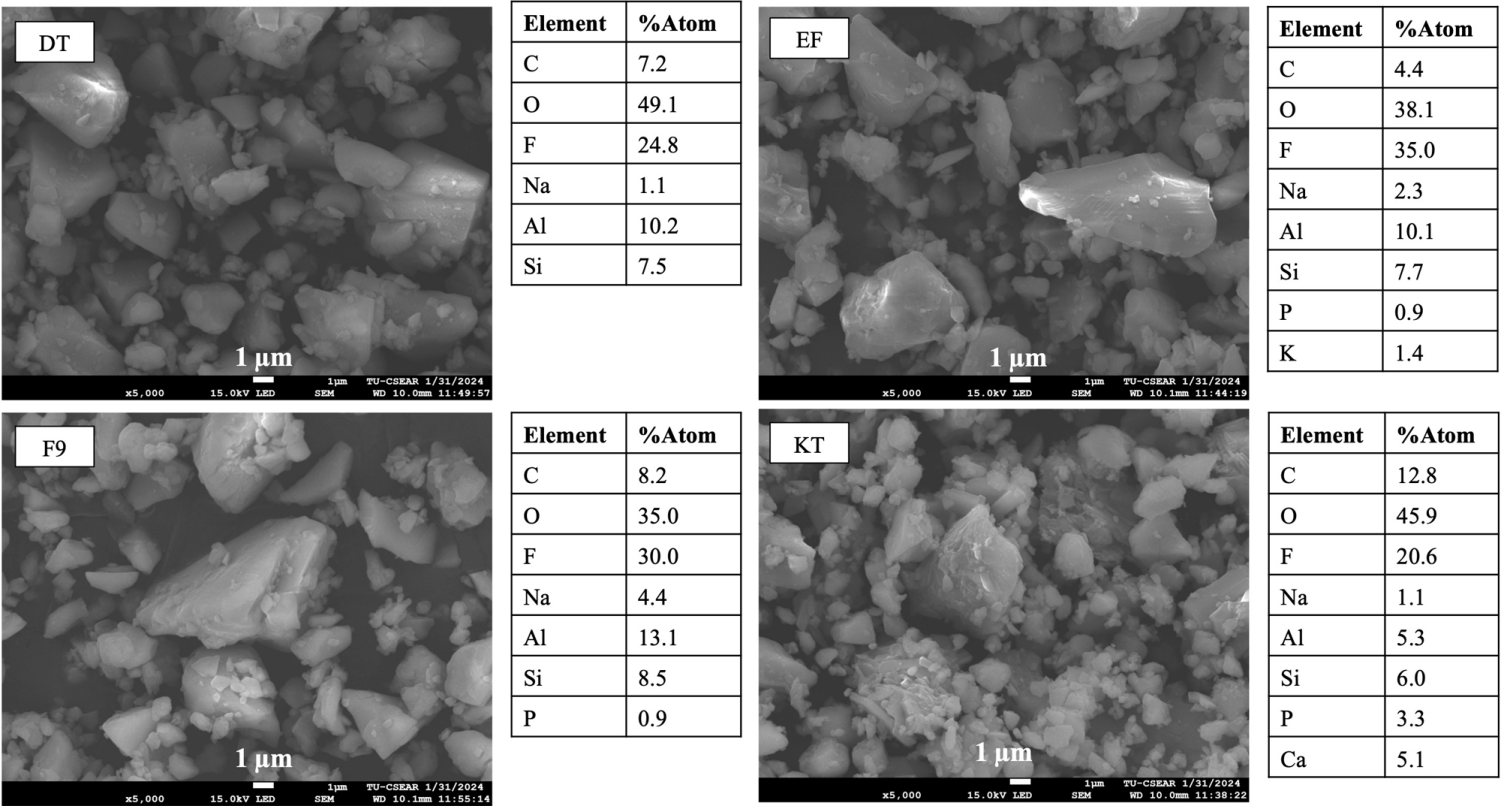
The SEM images of the powder phase and the elemental composition of each material

SEM images (Fig. 1) showed that the powder phase of all materials consisted of a mix of small and large particle diameters. Additionally, EDX results indicated higher fluorine content (atomic%) in EF and F9 compared to DT and KT. The average fluorine content (mean ± SD) measured from 3 points of each material was 23 ± 2% for DT, 30 ± 4% for EF, 27 ± 7% for F9, and 11 ± 9% for KT.

### Setting kinetics

**Fig. 2:**
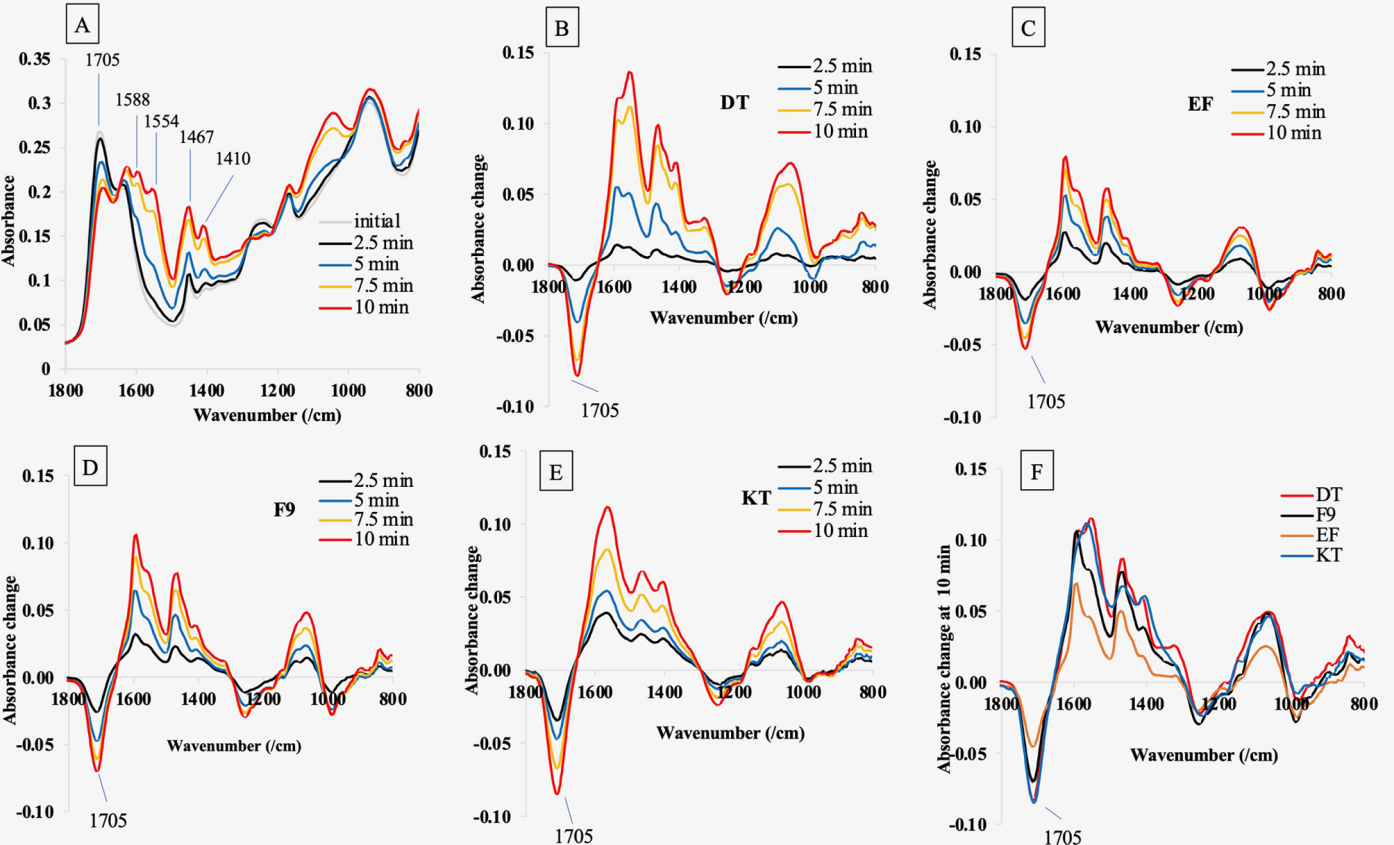
(**A**) Representative FTIR spectra of DT, (**B**-**E**) changes of spectra of all materials, and (**F**) the changes of spectra for all cement at 10 min. Difference spectra were calculated by subtracting the initial spectrum from that at later times. The reduction of the peak at 1705 cm^–1^ due to the neutralization of the polyacid was used to calculate the reaction extent

Example FTIR spectra versus time after mixing are provided in Fig. 2A. During the set, GICs showed a decrease in peak absorbance at 1705 cm^–1^ due to the loss of the carboxylic acid group (COOH) and an increase in multiple polyacrylate salt peaks at lower wavenumbers. The pattern of changes obtained from all materials was similar, which was presented by a representative sample from DT. Difference spectra (obtained by subtracting the initial spectrum from those at later times) for all materials given in Fig. 2B-D. Whilst with DT (Fig. 2B), there was a delay before significant absorbance change, with all other cements, no delay was observed. The relative intensities of different salt peaks showed only minor variations with time for a given cement but could vary between cements. For example, difference spectra for DT and KT at 10 min demonstrated greater absorbance change at 1554 and 1410 cm^–1^ than F9 and EF (Fig. 2F).

**Fig. 3:**
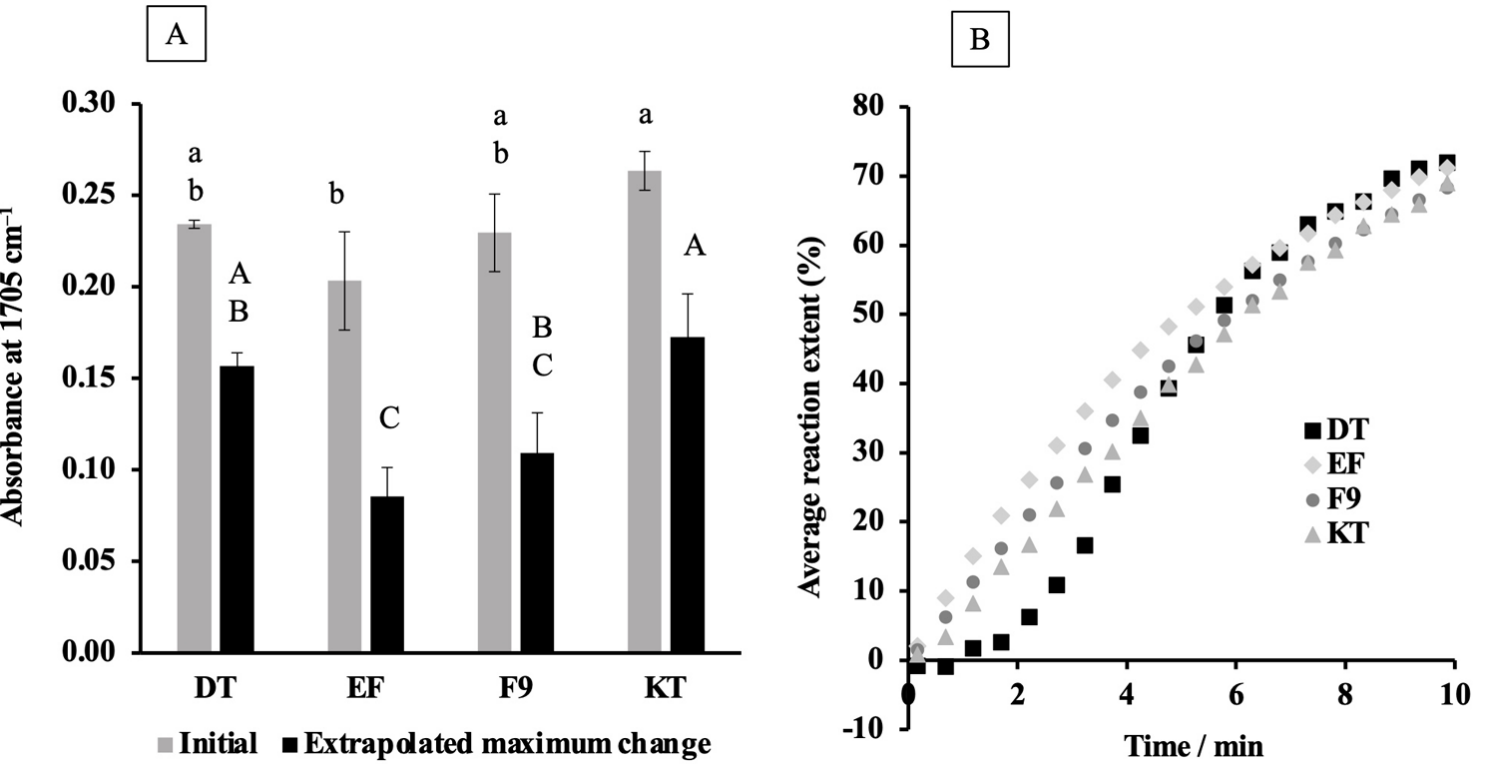
(**A**) Average of initial absorbance change at 1705 cm^–1^ and the maximum extrapolated absorbance change at 1705 cm^–1^. Data are mean and 95% CI (*n* = 3). The same lower-case and upper-case letters indicated *p* < 0.05 for the initial and extrapolated maximum change, respectively. (**B**) The changes in average reaction extent over time obtained from a representative sample of each group

The average initial absorbance at 1705 cm^–1^ was similar for DT, F9, and EF (*p* > 0.05), suggesting comparable initial levels of polyacid (Fig. 3A). However, the initial absorbance of KT was significantly higher than EF (*p* = 0.013). Maximum extrapolated absorbance change following reaction, however, was greater for DT and KT than for F9 and EF (Fig. 3A). The extrapolated absorbance change of EF was significantly lower than both DT (*p* = 0.006) and KT (*p* = 0.002). Using the 1705 cm^–1^ acid peak, a clear delay in the reaction can be observed for DT followed by a rapid reaction between 3 and 5 min (Fig. 3B). For all other cements, there was a steady reaction up to 5 min. The reaction rate slowed for all cements after 5 min. Reaction levels at 10 min were ~ 70% of extrapolated final values in all cases.

### Biaxial flexural strength (BFS) and modulus (BFM)

**Fig. 4:**
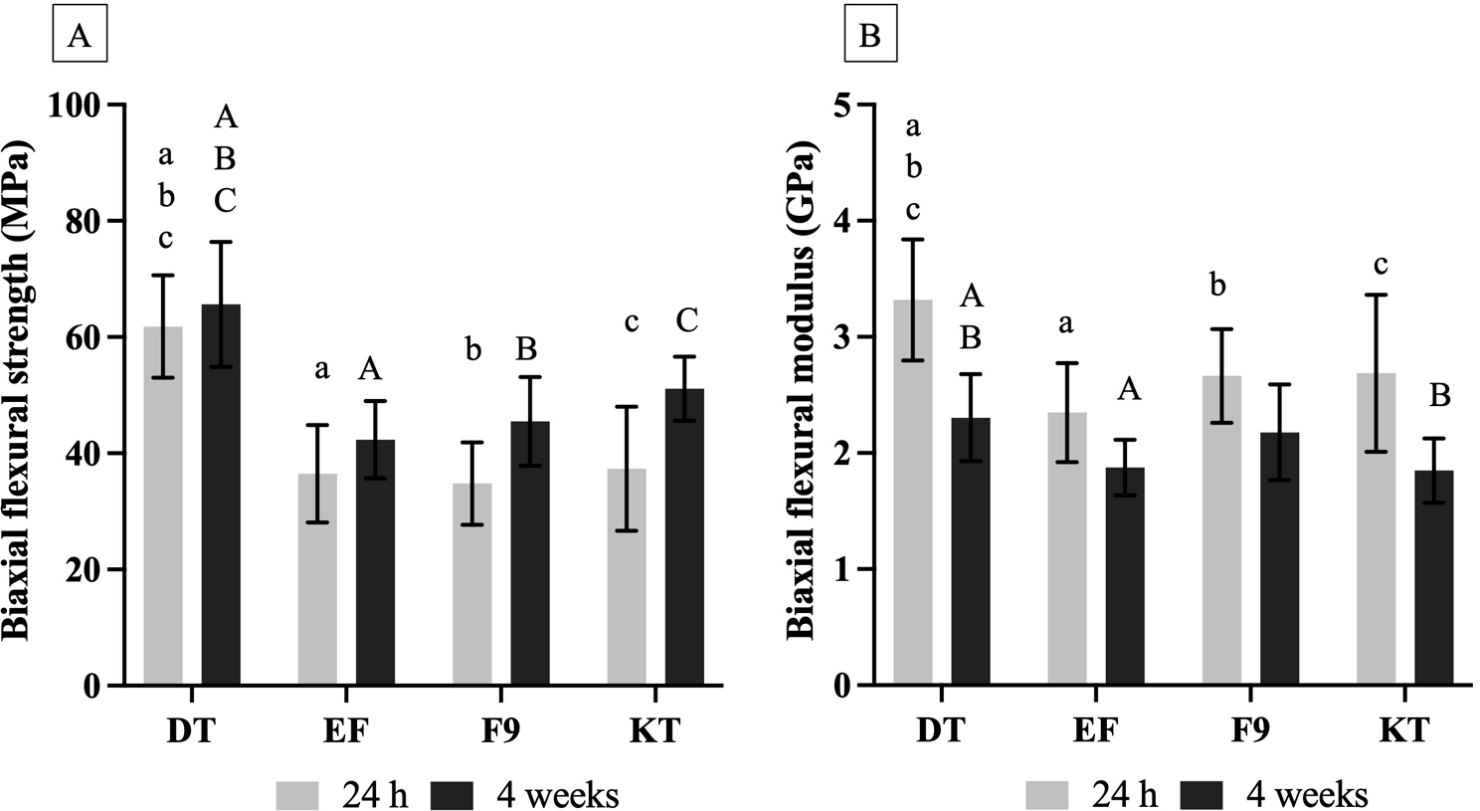
(**A**) Biaxial flexural strength and (**B**) biaxial flexural modulus after 24 h and 4 weeks of immersion in deionized water. Data are mean and 95% CI (*n* = 10). The same lower-case and upper-case letters indicate *p* < 0.05 for results at 24 h and 4 weeks, respectively

At 24 h (Fig. 4A), the highest BFS was observed for DT (62 ± 6 MPa). Other materials were statistically similar (*p* > 0.05), presenting significantly lower BFS in comparison to DT (*p* < 0.01). This trend continued at 4 weeks, with all groups showing a significant increase in BFS after storage (*p* < 0.05).

For BFM at 24 h (Fig. 4B), DT also showed the highest value (2.4 ± 0.4 GPa)(*p* < 0.05). No significant differences were detected between EF, F9, and KT (*p* > 0.05). After 4 weeks, the BFM of all materials increased significantly (*p* < 0.05). The BFM of DT (2.7 ± 0.4 GPa) remained significantly higher than that of EF and KT (*p* < 0.05) but was not significantly different from F9 (2.2 ± 0.4 GPa)(*p* = 0832).

**Fig. 5:**
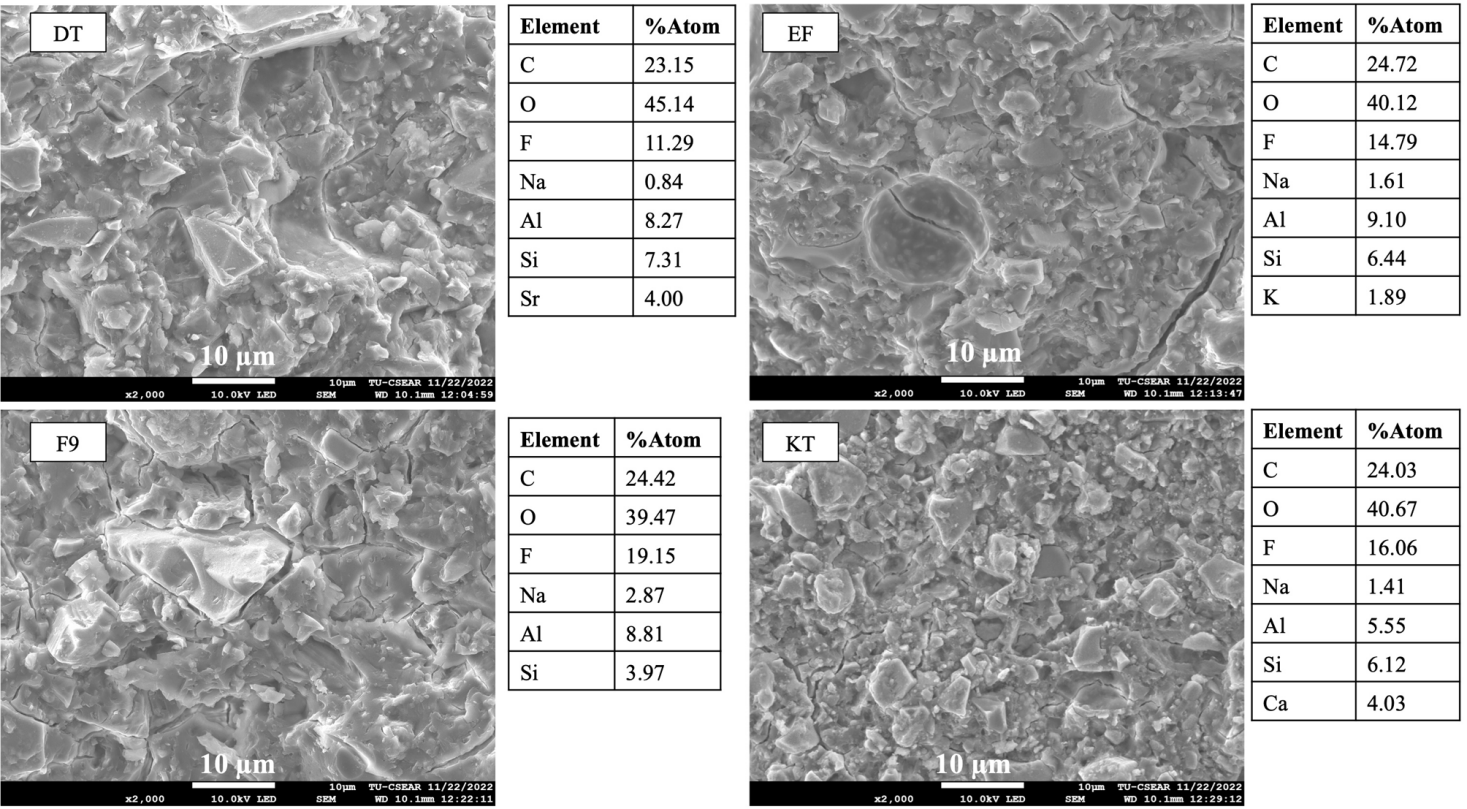
The SEM images and EDX analysis of fracture surfaces from representative samples of each material after BFS testing at 4 weeks

Fracture surfaces of the representative specimen from each material showed the remaining glass fillers in the matrix (Fig. 5). EDX analysis indicated that multiple elements such as F, Si, and Al were detected on the fracture surfaces in all materials.

### Surface microhardness

**Fig. 6:**
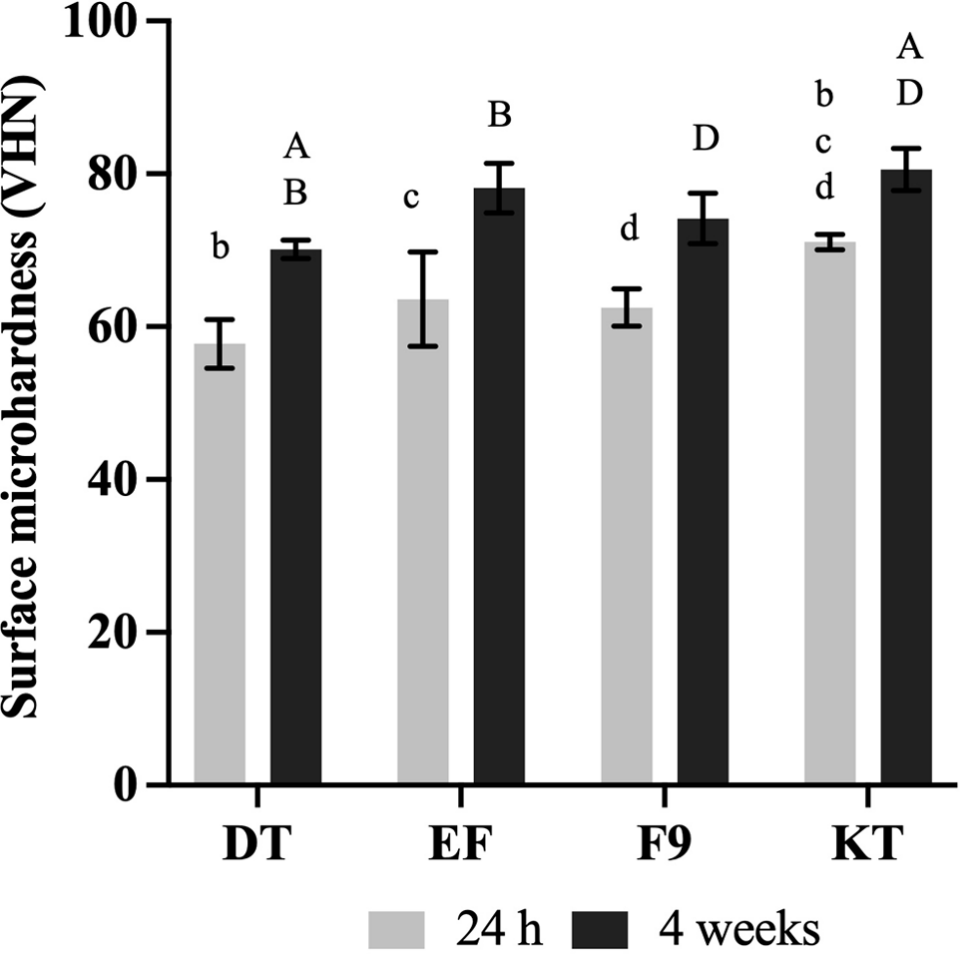
Vickers surface microhardness after 24 h and 4 weeks of immersion in deionized water. Data are mean and 95% CI (*n* = 5). The same lower-case and upper-case letters indicate *p* < 0.05 for results at 24 h and 4 weeks, respectively

At 24 h (Fig. 6), KT exhibited the highest surface microhardness (71.1±1.0 VHN), which was significantly higher than DT (57.8±3.2 VHN), EF (63.6±6.1 VHN), and F9 (62.5±2.4 VHN) (*p* < 0.05). At 4 weeks, surface microhardness values of all materials increased significantly (*p* < 0.05). KT maintained the highest value (80.6 ± 2.8 VHN), which was significantly greater than both DT (70.1 ± 1.2 VHN) and F9 (74.5 ± 3.3 VHN) (*p* < 0.01).

### Ion release

**Fig. 7:**
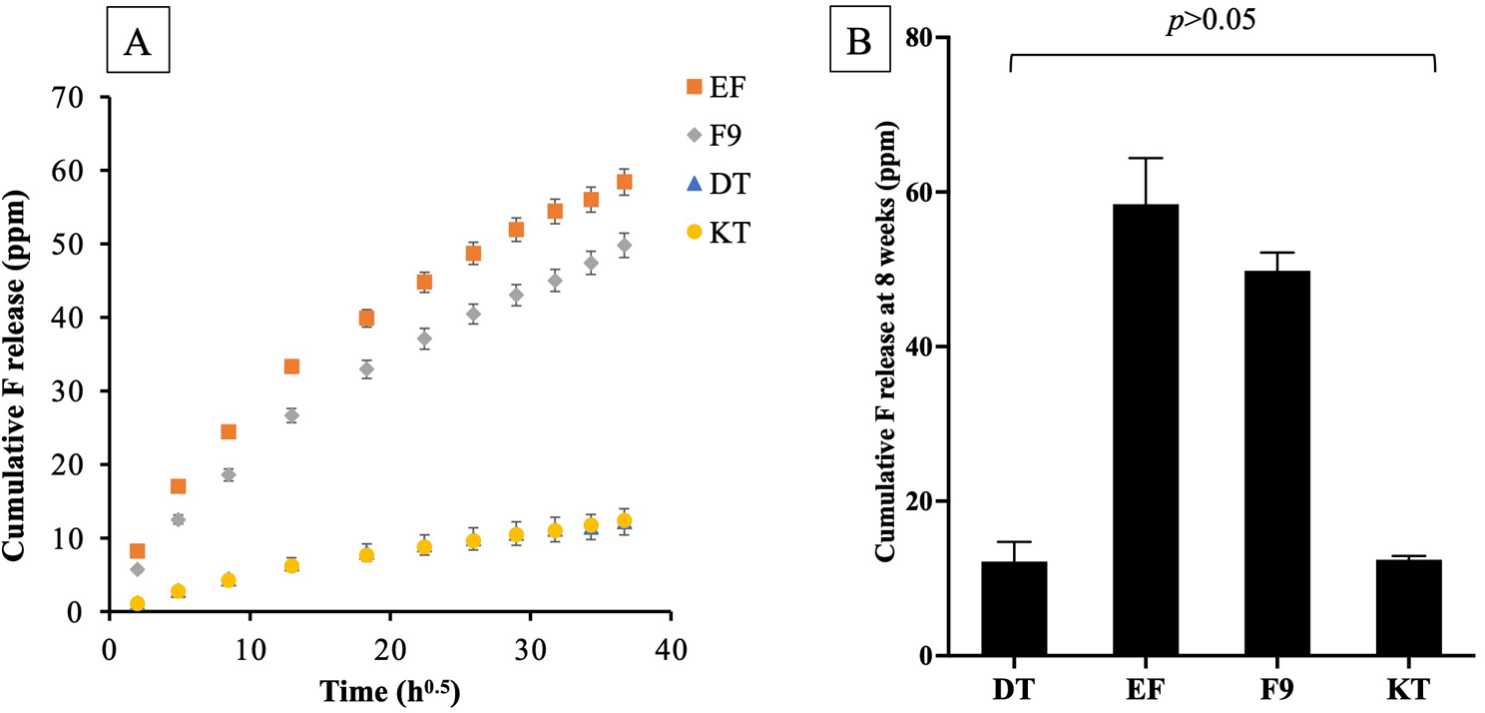
(**A**) Fluoride release versus square root of time in hours upon immersion in deionized water for 8 weeks. (**B**) The cumulative release of fluoride at 8 weeks for each material. Data are mean and 95% CI (*n* = 5). Line indicated *p* > 0.05

An initial burst release of fluoride was observed at 24 h (Fig. 7A). EF and F9 showed a continual increase in fluoride release over the 8-week measurement period. In contrast, for DT and KT, the increase was slower, beginning to level off at approximately 1 week. The highest cumulative fluoride release at 8 weeks (Fig. 7B) was from EF (58.4 ± 4.2 ppm), which was significantly higher than F9 (49.8 ± 1.7 ppm), DT (12.2 ± 1.8 ppm), and KT (12.4 ± 0.3 ppm) (*p* < 0.05). The fluoride release from DT was comparable to that of KT (*p* = 0.990).

EF and F9 also exhibited higher releases of Al and P than DT and KT (Table 3). Additionally, DT showed a significantly higher release of Al, P, and Sr compared to KT (*p* < 0.05).

**Table 3:** The mean (95% CI) of the concentration of elements after immersion in deionized water for 8 weeks. The same letters in each row indicate a significant difference between materials (*p* < 0.05)

Material/element (µg/L)	DT	EF	F9	KT
Al	540 ^a^ (126)	19,858 ^a^ (2,078)	10,592 ^a^ (1,163)	268 ^a^ (17)
P	716 ^b^ (13)	1,786 ^b^ (131)	1,300 ^b^ (79)	684 ^b^ (10)
Sr	416 ^c^ (66)	292 ^c^ (23)	190 ^c^ (26)	112 ^c^ (4)

### Cytotoxicity

**Fig. 8:**
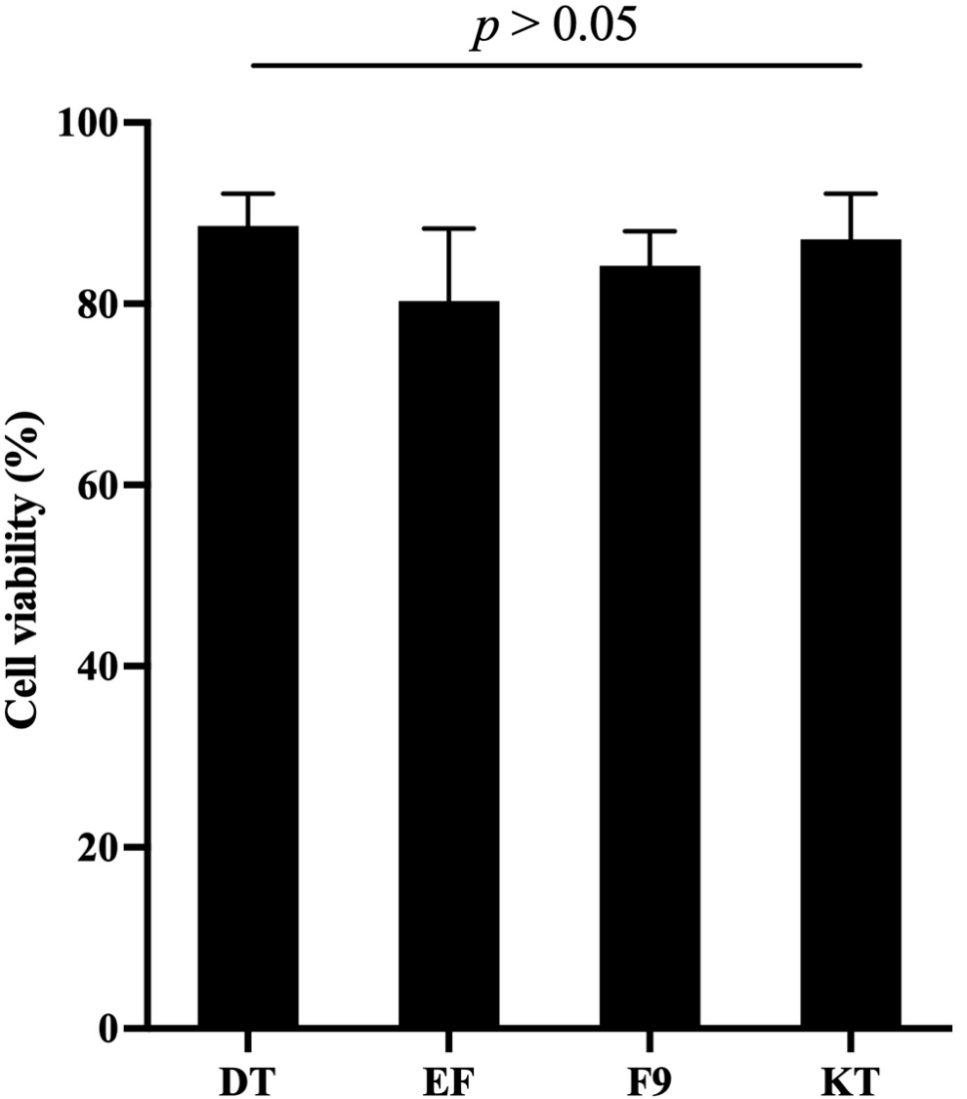
Percentage of cell viability of mouse fibroblast after exposure to extract from specimens of each material. Data are mean and 95% CI (*n* = 10). The line indicates *p* > 0.05

The highest cell viability of mouse fibroblast after exposure to extract from each material was observed from DT (88 ± 4%), followed by KT (87 ± 4%), F9 (84 ± 3%), and EF (80 ± 6%) as shown in Fig. 8. However, no significant difference was detected among the materials (*p* > 0.05).

## Discussion

This study compared the physical and mechanical properties of novel conventional elastomeric micelles-containing GICs (DT) with other commonly used materials. The results indicated that DT had significantly higher biaxial flexural strength than the other materials but exhibited lower fluoride release than EF and F9. Therefore, the null hypothesis was rejected.

The setting reaction of glass ionomer cement (GICs) is based on an acid-base reaction between polyacrylic acid and the fluoroaluminosilicate glass in the cement powder [36]. Therefore, a reduction in the FTIR peak representing the acid group in the spectroscopic analysis was used as an indicator of the acid reacting with the glass to form polyacrylate salts [25]. It was expected that the high level of acid-base reaction could potentially promote strong crosslinking of glass-matrix interaction, ultimately enhancing the mechanical properties of GICs [37, 38]. A slight delay in the inhibition of the reaction (approximately 2 min after the start of data collection) was observed for DT, which could be attributed to the effect of tartaric acids [11]. These acids may act as intermediates, forming tartrate salts before the final reaction between their anions and the carboxylic groups of the polyacrylic acid [36]. This inhibition time could provide a working time for GIC to be placed and adapted within the prepared cavity before a rapid snap setting [39]. However, it should be mentioned that the manufacturers did not reveal the actual composition of each product. Therefore, a direct comparison may not be possible.

This study assessed the setting reaction of GICs for up to 10 min. Hence, the setting kinetics observed in this study may only be suitable for assessing the kinetics during the hardening times (1.5–6 min) required by BS EN ISO 9917-1 2007 Dentistry-Water-based cements [40]. From a clinical perspective, faster reaction kinetics may indicate a quick setting for GIC, potentially reducing the susceptibility to fracture due to early mastication on the restoration [41]. However, the setting reactions of GICs can continue for several months [42]. This could be, therefore, considered a limitation of the current study, requiring future tests to determine the setting kinetics over longer time points.

The high flexural strength value would help ensure the suitability of GICs for placing in load-bearing cavities such as Class II restorations in posterior teeth. While there is currently no specific standard for the minimum flexural strength of GICs, the ISO 4049:2019 (Dentistry-Polymer-based restorative materials) requires a minimum flexural strength of 80 MPa for polymer-based restorative materials intended for permanent restorations in occlusal areas [43]. None of the GICs in this study met this recommended threshold. This may imply that these materials should be primarily used in conservative load-bearing cavities or Class I atraumatic restorative treatment (ART) restoration [44–46]. DT exhibited the highest BFS at 24 h (~ 62 MPa), which was slightly higher than previously reported ranges of 45–58 MPa from the 3-point bending test [23]. This discrepancy could be attributed to different testing protocols. Biaxial flexural strength testing was reported to show higher strength and greater reliability compared with the 3-point bending test [47]. This could be due to the uniform stress distribution, easy control in preparing small specimens, increased survival at a given load due to smaller volume, and narrow defect distribution [47, 48].

The highest BFS of DT could be due to its higher powder-to-liquid ratio (4.9:1) compared with other materials (3.0:1 to 3.6:1) [35]. Another explanation could be that the elastomeric micelles may help seal or delay the crack propagation. However, the effects of delayed crack propagation should be confirmed in future work using fracture toughness or fatigue testing [49]. The lack of assessment of mechanical behavior upon cyclic loading or fatigue testing is a limitation of the current study. The fatigue test may be more relevant to confirm the crack-inhibiting actions for DT because the restorations were subjected to a sub-critical repeated loading [50].

An increase in flexural strength and surface microhardness was detected in all GICs upon immersion in water. This may be due to the maturation process of the setting reaction of GICs [42]. The reduction in modulus of elasticity could be due to the dissolution of glass, which may reduce the rigidity of the material [35]. Another possibility could be the increase in unbound water, which could act as a plasticizer, reducing the rigidity of the polymer matrix in GICs [51].

EF and F9 showed higher fluoride release compared to other materials. This was in accordance with a previous study [52] and a consensus that ranked EF and F9 as the first and third materials of choice among conventional GICs for long-term restorative materials [53]. The level of fluoride release was governed by several key factors, such as the composition of the glass, the porosity and solubility of materials, the molecular weight of a polyacid, and the powder-to-liquid ratio [35, 54, 55]. EDX analysis of the powder phase showed that EF and F9 contained higher levels of fluoride compared with other materials., which may partly explain the higher level of fluoride release observed with EF and F9. This result was obtained from a single-point EDX analysis from the glass particles, which should be interpreted with caution.

The releasing profile of fluoride from GICs in this study was in accordance with a diffusion-controlled pattern reported in the previous study [56]. This pattern usually consists of an initial burst release at ~ 24 h, which was attributed to the rapid washout of fluoride from the cement occurring during the initial setting phase [57]. Then, acid-base reactions continue at a slower rate, resulting in a sustained release of fluoride at low levels that could last from several months up to 3 years [58, 59]. A limitation of this study was that the duration of fluoride and other elemental releases was monitored for only approximately 2 months. It was expected that the GICs would continue to exhibit ion-release actions over time. Future studies should investigate both ion-releasing and rechargeability [60] of the materials over extended periods (e.g., 12–36 months) to assess their long-term remineralizing potential.

It should be mentioned that the minimum requirement for fluoride release to promote clinical benefits for caries management from the ISO standard for GICs has not been established. It was, however, suggested that fluoride at a concentration of 0.03–0.7 ppm could inhibit mineral loss and enhance remineralization in dentin [10]. This may be associated with the reduction of *S. mutans* and inhibits the bacteria from metabolizing carbohydrates [55, 61]. Furthermore, all tested materials provided elemental release other than fluoride, such as Al, Sr, and P. A high level of elemental release was generally observed in EF. According to the manufacturer, the powder phase of EF contains a mixture of large and small particles of highly reactive fluoroaluminosilicate glass, referred to as a “glass hybrid material” [62]. This highly reactive glass may increase interaction with acids, resulting in a greater level of elemental release. The release of multiple elements was expected to enhance the bioactive properties of GICs, such as promoting mineralization or providing buffering effects [11, 63]. For example, the replacement of Ca with Sr in glass ionomer cement showed an increase in the radiopacity of the material and enhanced tooth remineralization [64, 65]. Sr was also believed to exhibit synergistic effects with fluoride in controlling dental biofilm [66]. It should be noted that Sr was not detected in the powder phase by the EDX, which could be due to the low concentration of the element in the glass network compared with other elements.

The conventional glass ionomer cement added with elastomeric micelles demonstrated cell viability comparable to that of commonly used commercial materials. The percentage of viability of all materials was greater than 70% relative to the blank control. This may suggest that all materials complied with the requirement of ISO 10993-5:2009, Biological evaluation of medical devices Part 5: Tests for in vitro cytotoxicity [31]. According to the standard, a reduction in cell viability greater than 30% is considered cytotoxic [31]. The results from the current study also correlated with the previous study, which indicated the good biocompatibility of conventional GICs to both L929 mouse fibroblasts and human dental pulp cells [67]. A satisfactory cytocompatibility was usually observed with conventional GICs compared with resin-modified GICs due mainly to the lack of toxic components such as 2-hydroxyethyl methacrylate [11, 68, 69].

The Minamata Convention advocates for the phasing down of dental amalgam to mitigate the environmental risk of releasing mercury waste into the environment [70]. The available alternative direct restorative materials, such as GICs, have improved over time but still exhibit limited strength and questionable long-term performance [71]. The current study demonstrated that GIC added with elastomeric micelles exhibited satisfactory mechanical properties and ion-releasing actions, suggesting its potential as an alternative GIC material. However, it is important to mention that this is an in vitro study. Therefore, clinical studies are necessary to assess the long-term effectiveness of this material compared with other GICs or resin composite in clinical settings.

## Conclusions

The novel glass ionomer cement containing elastomeric micelles (DT) exhibited similar levels of acid-base neutralization, surface microhardness, and cytotoxicity as compared to other commercial materials. However, DT has demonstrated superior strength. While DT exhibited lower levels of fluoride release than the commonly used GICs, it still falls within the range observed with a commercial comparison. These findings suggest that DT could be considered a viable option for load-bearing restorations where conventional GIC is deemed suitable.

## Data Availability

The datasets used and/or analyzed during the current study available from the corresponding author on reasonable request.

## Abbreviations

ART: Atraumatic restorative treatment
ATR-FTIR: Attenuated total reflectance-Fourier transform infrared spectroscopy
BFS: Biaxial flexural strength
BFM: Biaxial flexural modulus
DT: Deltafil
EDX: Energy Dispersive X-Ray Analysis
EF: EQUIA Forte HT Fil
F9: Fuji IX GP Extra
GICs: Glass ionomer cements
ICP-OES: Inductively coupled plasma-optical emission spectrometry
KT: Ketac molar
MTT: 3-[4,5-dimethylthiazol-2-yl]-2,5 diphenyl tetrazolium bromide
PEG: Polyethylene glycol
PU: Polyurethane
SEM: Scanning electron microscopes
VHN: Vickers hardness number
